# Radar in 7500 m Well Based on Channel Adaptive Algorithm

**DOI:** 10.3390/s25195994

**Published:** 2025-09-28

**Authors:** Handing Liu, Huanyu Yang, Changjin Bai, Siming Li, Cheng Guo, Qing Zhao

**Affiliations:** School of Resources and Environment, University of Electronic Science and Technology of China, Chengdu 611731, China; 202321070101@std.uestc.edu.cn (H.L.); yhy875356263@gmail.com (H.Y.); 202222070301@std.uestc.edu.cn (C.B.); 202422070302@std.uestc.edu.cn (S.L.); guocheng@uestc.edu.cn (C.G.)

**Keywords:** borehole radar, deep-well telemetry, SSTDR, adaptive equalization, LMS, OFDM, least-squares channel estimation

## Abstract

Deep-well radar telemetry over ultra-long cables suffers from strong frequency-selective attenuation and impedance drift under high temperature and pressure. We have proposed a channel-adaptive “communication + acquisition” architecture for a 7500 m borehole radar system. The scheme integrates spread-spectrum time domain reflectometry (SSTDR; m-sequence with BPSK) to monitor the cable in situ, identify termination/cable impedance, and adaptively match the load, thereby reducing reflection-induced loss. On the receiving side, we combine time domain adaptive equalization—implemented as an LMS-driven FIR filter—with frequency domain OFDM equalization based on least-squares (LS) channel estimation, enabling constellation recovery and robust demodulation over the distorted channel. The full processing chain is realized in real time on a Xilinx Artix-7 (XC7A100T) FPGA with module-level reuse and pre-stored training sequences for efficient hardware scheduling. In a field deployment in the Shunbei area at 7500 m depth, radar results show high agreement with third-party geological logs: the GR-curve correlation reaches 0.92, the casing reflector at ~7250 m is clearly reproduced, and the key bottom depth error is 0.013%. These results verify that the proposed system maintains stable communication and accurate imaging in harsh deep-well environments while remaining compact and implementable on cost-effective hardware.

## 1. Introduction

Data telemetry is one of the most critical technologies in deep well exploration [1]. Cables are preferred due to their corrosion resistance, wear resistance, and ability to withstand greater tension. The transmission channel between surface and downhole instruments typically uses armored cables. However, in actual logging operations, testing instruments are often placed in harsh environments more than 6000 m below the surface [2]. The environmental temperature exceeds 120 degrees Celsius, and the pressure exceeds 140 megapascals [3]. Unfortunately, long-distance cables cause severe attenuation of the high-frequency components of the signal, leading to signal distortion. The harsh working environment and channel characteristics pose challenges to the accuracy and stability of logging. In the 20th century, people explored communication solutions for cable transmission. They experimented with coding and modulation communication technologies, which significantly improved communication rates [4]. In the 1960s, pulse code modulation (PCM) encoding methods were adopted, achieving a communication rate of 8 kbps, which was a major breakthrough at the time. By the 1980s, more complex encoding techniques were being explored for logging transmission systems. For example, a wellbore transmission system using Manchester encoding achieved a communication rate of 20 kbps [5]. During the same period [6], Xerox Corporation explored binary phase shift keying (BPSK) schemes, achieving a communication rate of 100 kbps for the first time. This marked the beginning of research into communication modulation and demodulation in downhole wired communication systems.

In 2005, the EILOG system developed by China Petroleum Logging Company introduced coded orthogonal frequency division multiplexing (C-OFDM) modulation technology, enabling it to achieve a rate of 400 kbps over a 7000 m-long cable [7]. In 2021, a team from the University of Science and Technology of China achieved a speed of 1.4 Mbps over a 7000 m cable by employing OFDM modulation technology [8]. This demonstrates the significant role of OFDM in cable transmission.

However, despite the fact that OFDM modulation technology has enabled communication speeds of up to 1.4 Mbps over extremely long cables, underground communication systems must still consider the impact of the complex and variable underground environment on equipment. Current improvements to deep well remote transmission systems primarily focus on algorithmic enhancements [9]. The team of University of Science and Technology of China optimized computational modules including symbol synchronization, frequency domain equalization, data compression, and sampling clock offset compensation. This enables stable transmission of 1.4 to 2.3 Mbps data over 7000 m of armored logging cable. Additionally [10], some researchers have efficiently analyzed the propagation characteristics of electromagnetic waves in oil wells using pattern matching technology (MMT), thereby optimizing the design of wireless telemetry systems. Additionally, there are innovations focused on image processing to enhance communication quality [11,12,13].

Spread Spectrum Time Domain Reflectometry (SSTDR) is adopted as the detection means of the cable. At the same time, adaptive time domain equalization and frequency domain equalization are selected as the signal restoration means, which can effectively resist the OFDM signal distortion caused by the cable in the well. By multiplexing the detection signal and training signal, the bandwidth can be saved and the overall system redundancy can be reduced. Multiple adaptive means can be combined organically. The program can be used to monitor the status of cables in wells. Therefore, the application of cable monitoring technology in the field of well logging is of great significance to the research of well logging technology.

Most of the current research on impedance matching and condition monitoring of cables has been focused on the fields of power supply and transportation and has started relatively recently. Some of the main techniques used to measure cables are reflection techniques, such as Time Domain Reflectometry (TDR). The main form of this technique is by transmitting a test signal into the cable. The reflected signals are collected and analyzed to estimate the state of the cable [14]. As early as 1931, Rohrig, a researcher in the United States, first applied TDR to the localization and identification of defects in cable lines in the power and telecommunication industries [15]. In 2005, Paul Smith and Cynthia Furse, researchers at the University of Utah et al., proposed the Extended Spectrum Time Domain Reflectometry method. This scheme uses pseudo-random sequences with carrier modulated signals as test signals shot on aircraft cables for testing, which verifies the feasibility of the SSTDR cable fault on-line detection method [16].

Research on cable communication in deep earth logging has been developed over a period of time. However, with the expanding needs of oilfield exploration, especially in the deep earth logging environment, its stability is difficult to withstand the test of complex and harsh environments. In order to make up for this defect, we introduce adaptive communication technology to ensure reliable communication and improve communication quality under various environmental conditions.

## 2. Fundamentals of Adaptive Downhole High-Speed Communication

### 2.1. Channel Characteristics of Cables

**Figure 1:**
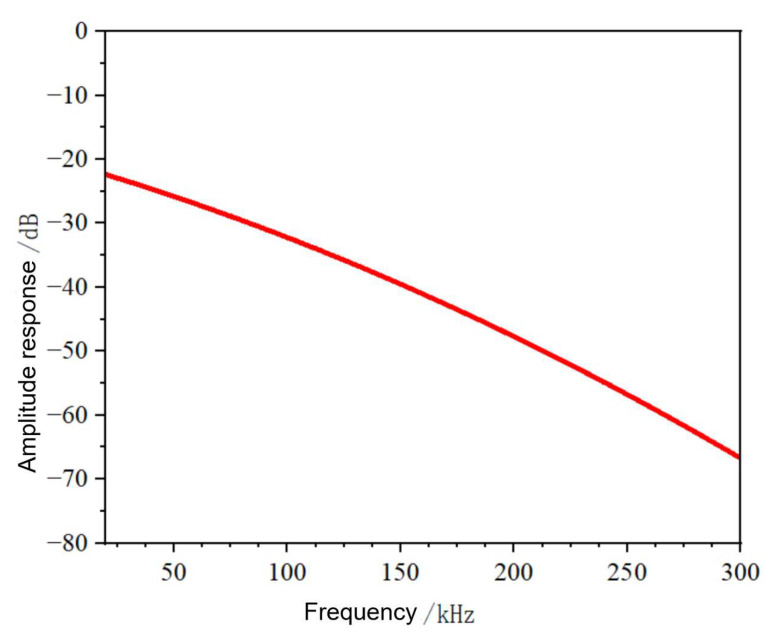
Amplitude–frequency response diagram of seven-core armored cable.

In order to investigate the relationship between frequency and cable channel gain, the transmission characteristics of a seven-core armored logging cable of about seven kilometers were measured by a vector network analyzer. As can be seen in Figure 1, the attenuation of the amplitude response of the signal increases substantially with its frequency. The test results of the seven-core armored logging cable show that the attenuation of signals with higher frequencies is extremely high after transmission through the logging cable, so using signals with high frequencies as communication signals will lead to serious distortion of the final data received.

### 2.2. Spread Spectrum Time Domain Reflectometry

The difference between the spread spectrum time domain reflection method and the conventional time domain reflection method is that the test sequence needs to be modulated onto a sine wave and transmitted as a Binary Phase Shift Keying (BPSK) signal, which has the advantage of expanding the frequency spectrum and reducing the interference to the information transmission in the channel [17].

**Figure 2:**
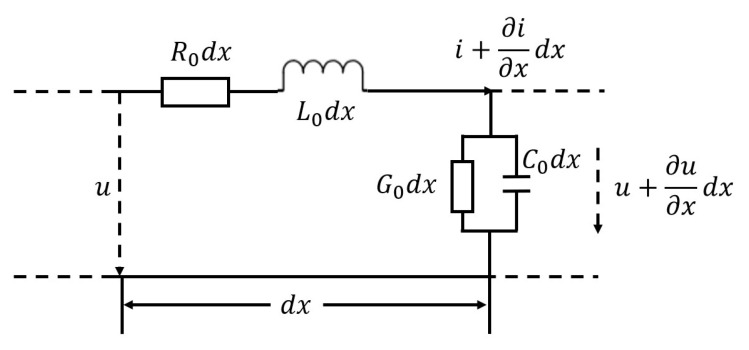
Parametric model of cable distribution.

In case of cable faults, the fault transient signals generated contain high-frequency components, which have shorter pulse widths than conventional voltage traveling waves, and their wavelengths are significantly smaller than the cable length, a characteristic that makes it impossible to analyze them according to the traditional centralized parametric model. Therefore, a distributed parameter circuit model is necessary to analyze such high-frequency fault signals, and the schematic diagram of the model is shown in Figure 2.

In this model, *R*_0_ denotes resistance per unit length, *L*_0_ denotes inductance per unit length, *C*_0_ denotes capacitance per unit length, and *G*_0_ denotes leakage conductance per unit length.

Based on Kirchhoff’s circuit laws, we can derive the basic equation for a transmission line:

(1) $$\left\{\begin{array}{c} \frac{\partial u}{\partial x}+L_{0}\frac{\partial i}{\partial t}+R_{0}i=0\\ \frac{\partial i}{\partial x}+C_{0}\frac{\partial u}{\partial t}+G_{0}i=0 \end{array}\right.$$

where *i* and *u* in Equation (1) are the current and voltage along the transmission line. In turn, by taking a partial derivative with respect to *t* and with respect to *x*, we obtain the following:

(2) $$\left\{\begin{array}{c} \frac{\partial^{2}u}{\partial x^{2}}=L_{0}C_{0}\frac{\partial^{2}i}{\partial t^{2}}+\left(R_{0}C_{0}+G_{0}L_{0}\right)\frac{\partial i}{\partial t}+R_{0}G_{0}i\\ \frac{\partial^{2}i}{\partial x^{2}}=L_{0}C_{0}\frac{\partial^{2}u}{\partial t^{2}}+\left(R_{0}C_{0}+G_{0}L_{0}\right)\frac{\partial u}{\partial t}+R_{0}G_{0}u \end{array}\right.$$

Obtain the generalized solution of the equation as follows:

(3) $$\left\{\begin{array}{c} u\left(x,t\right)=f_{1}\left(t-\frac{x}{\nu }\right)+f_{2}\left(t+\frac{x}{\nu }\right)=u^{+}\left(t-\frac{x}{\nu }\right)+u^{-}\left(t+\frac{x}{\nu }\right)\\ i\left(x,t\right)=i^{+}\left(t-\frac{x}{\nu }\right)+i^{-}\left(t+\frac{x}{\nu }\right)=\frac{u^{+}\left(t-\frac{x}{\nu }\right)}{Z_{L}}-\frac{u^{-}\left(t+\frac{x}{\nu }\right)}{Z_{L}} \end{array}\right.$$

where ZL denotes the characteristic impedance of the cable, and *u+* denotes that the wave propagates in the positive direction of the X-axis with a speed of *v*. This shows that *u−* is the opposite, and from the generalized solution, it can be seen that there are fluctuations in the cable that propagate in the positive and negative directions of the X-axis at the same time, which reveal the phenomena of reflection and transmission of signals in the cable.

The characteristic impedance *Z*_L_ is calculated as follows:

(4) $$\left\{Z_{L}=\frac{u^{+}}{i^{+}}=\frac{u^{-}}{i^{-}}=\sqrt{\frac{R_{0}+j\omega L_{0}}{G_{0}+j\omega C_{0}}}\right.$$

The principle of cable fault detection is based on changes in the impedance characteristics of the cable; specifically, when a cable fails, its internal impedance characteristics will change. This phenomenon is based on the theory that electromagnetic waves are reflected when they encounter an impedance mismatch. Spread Spectrum Time Domain Reflection detects faults by using a specific modulated test signal that minimally interferes with the normal communication signals in the cable. Therefore, in the event of a fault, the test signal transmitted along the cable is reflected at the point of the fault and accompanied by the original signal back to the transmitting end, thus permitting precise localization of the fault location. Similarly, the full length of the cable as well as the total cable impedance information can be equivalently measured, depending on the end of the cable as a breakpoint.

#### Pseudo-Random Sequence

For the test signal, only with good autocorrelation and white noise characteristics can it realize online monitoring of cables and intelligent impedance identification, effectively guaranteeing the effectiveness of cable communication, so the selection of the test signal is also crucial. The spread spectrum time domain reflection method has strict requirements for the test sequence, and the characteristics of pseudo-random sequences satisfy the good correlation and white noise characteristics, which become the mainstream choice of the test sequence.

The m-sequence is formally similar to a random sequence, but is actually generated deterministically, so it has pseudo-random properties. At the same time, they are periodic; m-sequences have the longest periodicity among pseudo-random binary sequences, and the sequences do not repeat within a certain length. This makes them highly independent in communication and data transmission.

In this paper, ‘m-sequence’ denotes a maximum-length sequence (MLS)—a pseudo-random binary sequence generated by an m-stage linear feedback shift register (LFSR). We employ the MLS as the probing bit sequence because of its two-level balance and near-impulsive aperiodic autocorrelation, which facilitate synchronization and system identification.

The m-sequence has autocorrelation and smoothness, exhibits distinct peaks in the autocorrelation function, and shows a uniform distribution in the statistical properties. This characteristic enables it to achieve good signal separation and interference suppression in spread spectrum communication systems, thus simplifying the analysis and design process of signal processing. Therefore, the m-sequence is chosen as the test sequence in this experiment.

The pseudo-random sequence m-code consists of a string of periodic random numbers. The period is determined by the number of bits N of the shift register and the sequence is determined by the generating polynomial [18]. Where k is the number of register bits, Ck is the feedback coefficient for a k-stage flip-flop. The recursive formula, shift inputs, and the relationship between the states of the levels before the shift inputs are also included.

(5) $$a_{k}=\sum_{n=0}^{N}\left(C_{n}a_{\text{k-n}}\right)$$

A polynomial ƒ(x) is used to describe the feedback connection state of a linear feedback shift register:

(6) $$f(x)=\sum_{k=0}^{N}\left(C_{k}x^{k}\right)$$

**Figure 3:**
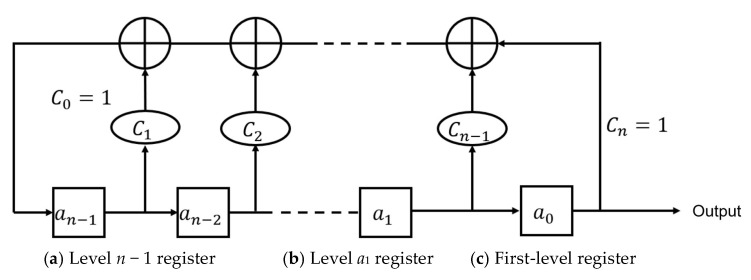
m-sequence generator schematic.

Usually, the m-sequence generator is called Simple Shift Register Generator (SSRG), and its general structure is shown in Figure 3. In the figure, each flip-flop (*n* = 1, 2, …k) constitutes a shift register, which represents the heteroscedastic operation, and C0, C1, C2, etc., and Ck are the feedback coefficients and the coefficients of the characteristic polynomial. The coefficients take the value of 1 to indicate that the feedback branch is connected and 0 to indicate that the feedback branch is disconnected.

### 2.3. BPSK Spread Spectrum Processing

SSTDR is a Time Domain Reflectometry method utilizing spread spectrum technique. In SSTDR, BPSK spread spectrum modulation technique is used to improve the signal’s immunity to interference and resolution.

The traditional time domain reflection method technique usually uses rectangular pulse signals or broadband signals for measurements, which have good resolution, but are susceptible to interference for long-distance transmissions or measurements in complex environments and are prone to interfering with communication signals [19]. In contrast, SSTDR avoids this situation by taking advantage of spread spectrum technology. BPSK spread spectrum modulation spreads a narrow band information signal by multiplying it by a relatively high frequency pseudo-random sequence to realize the spreading of the signal, which makes the signal’s frequency spectrum wider and better resistant to narrow band interference and multipath effects. As a result, the anti-interference capability of SSTDR is significantly better than that of conventional TDR technology in long-distance transmission and complex environments.

**Figure 4:**
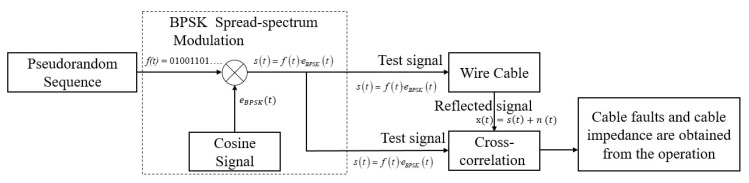
SSTDR schematic.

In addition, SSTDR utilizes the BPSK spread spectrum technology and can also achieve higher measurement resolution through the demodulation of the spread spectrum signal; it can realize a more accurate time measurement of the reflected signal, thus improving the accuracy and resolution of the measurement [20], and the schematic diagram of its method is shown in Figure 4.

The modulating signal in BPSK is usually a cosine signal and the e_BPSK_(t) expression is as follows:

(7) $$e_{BPSK}(t)=\cos(\Omega_{c}t+\theta_{0})$$

The pseudo-random sequence ƒ(x) is subjected to BPSK spreading in SSTDR, i.e., the modulation signal e_BPSK_(t) is used to multiply with the pseudo-random sequence in the time domain to obtain the spreading pseudo-random sequence expression s(t) as shown below:

(8) $$s\left(t\right)=f\left(t\right)\cdot e_{BPSK}\left(t\right)$$

#### Fault Detection Algorithm with Adaptive Impedance Matching

Where the detection signal enters into the cable and encounters a fault point, a reflection signal is generated to return to the DAC, and the delay time between the reflection signal and the detection (reference) signal collected by the FPGA is approximated as the round-trip time of the signal from the T-connector to the fault point. The collected reference signal x_1_(t) and x_2_(t) reflection signal are analyzed by a cross-correlation operation, where n_1_(t) and n_2_(t) are the noise carried by the reference and reflection signals, respectively, and s(t) is the original spread spectrum pseudo-random sequence signal.

(9) $$x_{1}\left(t\right)=s\left(t\right)+n_{1}(t)$$

(10) $$x_{2}\left(t\right)=s\left(t\right)+n_{2}(t)$$

The upper computer system performs the cross-correlation operation with the collected data, and its autocorrelation calculation formula is as follows:

(11) $$R_{12}\left(t\right)=E\left[x_{1}\left(t\right)x_{2}\left(t\right)\right]=\alpha R_{ss}\left(\tau -p\right)$$

where x1(t): Reference (transmitted) signal; x2(t): Echo (received) signal; τ: Time delay variable for correlation calculations; p: Signal propagation delay round-trip to the fault point; α: Amplitude coefficient of the echo; Rss: Autocorrelation function of the probe sequence x(t); R12(τ): Cross-correlation function between the reference signal and the echo signal; and E: Expectation operator.

The distance to the fault is localized based on the delay characteristics of the reflected signal and the type of fault is identified by its amplitude characteristics. The location of the fault is calculated by analyzing the signal round trip time. In practice, we interpret the reflection-peak amplitude (after compensating for distance-related attenuation) to indicate the fault type. Open and short circuits produce the strongest peaks; they are distinguished by a 180° phase reversal observed in the waveform. Impedance mismatches yield moderate peaks whose strength increases with the severity of the mismatch, while distributed/partial faults often appear as multiple smaller peaks. Amplitude is thus a useful discriminator because larger impedance discontinuities generate stronger reflections. Assuming that the noise is additive ideal noise, its autocorrelation result can be regarded as αR_ss_(τ − p); the delay time is obtained from the peak moment of the inter-correlation, and the information of the effective length of the cable can be grasped by the delay time as well as the propagation speed of the signal in the cable.

**Figure 5:**
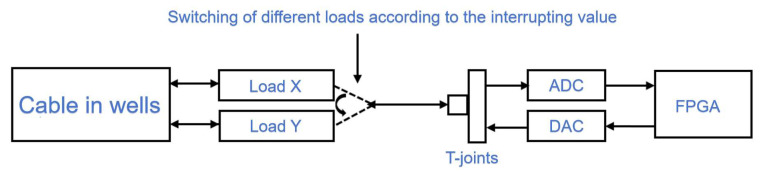
Schematic diagram of SSTDR impedance identification implementation program.

Since the open- or closed-circuit termination of a cable generates reflected signals, and the end of the cable is similar to an open circuit, SSTDR can evaluate the impedance characteristics of the cable termination by analyzing these reflections. The variation in the reflected signals from the start to the end of the cable allows the distributed impedance characteristics of the whole cable to be assessed, wherein the cable can be loaded with known and different load impedances based on this correlation value to the impedance of the cable under test, and the reflection signals obtained under different load impedances are operated with the correlation signals of the reference signals, the implementation of which is shown in Figure 5.

Where the autocorrelation peak of reference signal is $R_{ss}$. Inter-correlation peak of the reflected signal and reference signal $R_{12}$. ${max(R}_{12})$ is the maximum value of inter-correlation peak under different loads. ${min(R}_{12})$ is the minimum value of inter-correlation peak under different loads. Γ is the reflection coefficient with the following equation:

(12) $$\Gamma =\frac{2\left(R_{ss}-min\left(R_{12}\right)\right)}{\max\left(R_{12}\right)-min(R_{12})}-1$$

Through the reflection coefficient formula, the cable characteristic impedance estimation formula is obtained as follows. Z_L_ is the characteristic impedance of the cable and Z_0_ is the load impedance corresponding to R_ss_:

(13) $$\;\Gamma =\frac{Z_{L}-Z_{0}}{Z_{L}+Z_{0}}$$

(14) $$\;Z_{L}=\frac{R_{ss}-min\left(R_{12}\right)}{\max\left(R_{12}\right)-Rss}Z_{0}$$

From this, the target values of all SSTDRs for cable condition monitoring can be obtained, and the specific values of impedance values can be realized for the monitoring of abnormal cable conditions with real time. The specific types of reflection conditions during fault detection are as follows. When Z_0_ = Z_L_ and Γ = 0, the cable is normal. Where Z_L_ = 0, Γ = −1 is a short circuit fault. When Z_L_ = ∞, Γ = 1, is a high resistance fault. After obtaining the value of the cable characteristic impedance Z_L_, the value of the load impedance responsible for the ground end is adjusted by controlling the digital potentiometer through the FPGA.

### 2.4. Adaptive Time Domain Equalization

LMS adaptive filtering is a commonly used adaptive filtering technique, which is based on the statistical nature of the signal and is able to automatically adjust the filter parameters in order to reduce the signal distortion and improve the reliability of communication [21]. The algorithm usually acts in the error calculation and coefficient update module in digital logic circuits to update the weight coefficients of the FIR filter in real time [22].

#### 2.4.1. Digital Filtration

FIR filters can be used to filter signals from cables in wells. Conventional FIR filters usually have fixed coefficients, which makes it difficult to adapt to dynamically changing channels. The filter coefficients of the FIR filter module in the adaptive time domain equalization used in this system are dynamically adjustable to adapt to the changes in the cable channel. The output signal of the filter module can be expressed as follows:

(15) $$\;\;Y\left(n\right)=\sum_{k=0}^{N-1}w(k)X(n-k)$$

where $\;Y\left(n\right)$ is the output of the filter, w(k) is the weighting coefficients, and X(n−k) is the delay signal of the input signal. These weighting coefficients are adaptively adjusted using the LMS algorithm according to the actual conditions of the cable channel in the well to optimize the filtering effect.

#### 2.4.2. Error Calculation and Coefficient Update

The basic principle of the minimum mean square error is the most rapid gradient descent method [23]. But considering the difficulty of implementation on FPGA, the mean square error of the statistics is chosen to be estimated by the instantaneous deviation, which takes into account the accuracy and implementation complexity, and at the same time has a lower occupancy rate of resources. Considering the key indexes of accuracy, implementation complexity, and resource occupancy, the system finally adopts the LMS algorithm as the adaptive algorithm for time domain equalization.

The error calculation module is responsible for analyzing the error between the received cable signal and the reference signal and calculating the error signal to indicate the difference between the received signal and the desired signal. The error signal can usually be expressed as follows:

(16) $$e\left(n\right)\;=\;d\left(n\right)\;-\;y\left(n\right)$$

where is e(n) error signal, d(n) is the reference signal, and y(n) is the actual output of the filter. With the error signal, the loss function for its minimum mean square error can be calculated, and the loss function J for the minimum mean square error can be expressed as follows:

(17) $$\begin{array}{cc} J & =E\left(e^{2}\left(n\right)\right)\\ & =E[\left(d\left(n\right)-y\left(n\right){)}^{2}\right]\\ & =\;E\left[d^{2}\left(n\right)-2d\left(n\right)y\left(n\right)+y^{2}\left(n\right)\right]\\ & =\;E\left[d^{2}\left(n\right)\right]-2w^{T}P+w^{T}Rw \end{array}$$

And

(18) $$P=E\left[d\left(n\right)x\right]$$

(19) $$R=E\left[xx^{T}\right]$$

(20) $$x=\left[x\left(n+1-M\right)x\left(n+2-M\right)\ldots x\left(n\right)\right]$$

(21) $$w=\left[w_{1}w_{2}\ldots w_{M}\right]$$

The partial derivative of w with respect to P yields the following:

(22) $$\begin{array}{cc} \nabla \left(n\right) & =\frac{\partial E\left[e^{2}\left(n\right)\right]}{\partial w}\\ & =E\left[2e\left(n\right)\frac{\partial e\left(n\right)}{\partial w}\right]\\ & =2\left(Rw-2P\right) \end{array}$$

This leads to an iterative formula for the FIR filter weight coefficients that can be defined as follows:

(23) $$\begin{array}{cc} w\left(n+1\right) & =w\left(n\right)+c\left(-\nabla \left(n\right)\right)\\ & =\;w\left(n\right)+\mu \left(P-Rw\left(n\right)\right) \end{array}$$

The LMS algorithm replaces the mean square error with the square of the instantaneous error and obtains the gradient vector as follows:

(24) $$\nabla \left(n\right)=2e\left(n\right)\frac{\partial e\left(n\right)}{\partial w}=-2e\left(n\right)x\left(n\right)$$

Substituting into the iterative formula gives the following:

(25) $$w\left(n+1\right)=w\left(n\right)+2\mu e\left(n\right)x\left(n\right)$$

where w(n + 1) is the weight coefficient of the next time step, w(n) is the weight coefficient of the current time step, µ is the learning rate (step factor), e(n) is the error signal, and x(n) is the vector of input signals.

This adaptive filter for ultra-long cable communication in wells based on the LMS algorithm aims to improve the signal quality and reliability of cable communication systems in wells. By continuously and adaptively adjusting the weight coefficients of the filter, the filter can effectively reduce signal distortion, noise interference, and waveform distortion, thus making the communication more reliable and stable.

### 2.5. Adaptive Frequency Domain Equalization

The core of OFDM technology lies in the division of a wider channel into a number of achievable orthogonal subchannels, which means that the SNR of each subchannel needs to be predicted in advance by the receiver for the subsequent reduction process. Therefore, in order to obtain the SNR of each subchannel correctly, the transfer function of each of its channels needs to be estimated. Assuming that the number of subchannels in cable communication is N, the estimate of the transfer function of the kth subchannel can be expressed as follows:

(26) $${\hat{H}}_{k}=H_{k}+e_{k}$$

H_K_ is the actual transfer function of the kth subchannel and e_K_ is the error of the channel estimation. Assuming that the input signal is X_K_, then the estimate Ŷ of the output signal can be expressed as follows:

(27) $$\begin{array}{cc} {\hat{Y}}_{k} & ={\hat{H}}_{k}X_{k}+n_{k}\\ & =H_{k}X_{k}+e_{k}X_{k}+n_{k} \end{array}$$

In order to obtain an estimate of the channel, least square (LS) is used as the core algorithm for frequency domain equalization. Least square is a mathematical optimization technique used to obtain unknown data. It obtains an estimate of the channel by minimizing the square of the error and finding the best function match of the data [24].

Least square is also easier to implement in digital logic circuits due to the ability of FPGAs to process frequency domain data. Using the least square method, it is easy to find the unknown data and minimize the sum of squares of the errors between these found data and the actual data.

Assuming that the signal finally received at the kth subchannel is Y_k_, its signal not passing through the channel is X_k_, and the noise is denoted as n_k_, one obtains the following:

(28) $$Y_{k}=H_{k}X_{k}+n_{k}$$

Assuming that the transfer function estimate for this subchannel is Ĥ_k,LS_, the received to signal estimate Ŷ_K_ after X_K_ passes through the channel is as follows:

(29) $${\hat{Y}}_{k}={\hat{H}}_{k,LS}X_{k}$$

Minimize the error squared between Ŷ_K_ and Y_k_:

(30) $$\frac{\partial {\left(Y_{k}-{\hat{Y}}_{k}\right)}^{T}\left(Y_{k}-{\hat{Y}}_{k}\right)}{\partial {\hat{H}}_{k,LS}}=0$$

By bringing the received signal Y_k_ and the estimated value Ŷ_K_ into Equation (30):

(31) $$\frac{\partial {\left(Y_{k}-{\hat{H}}_{k,LS}X_{k}\right)}^{T}\left(Y_{k}-{\hat{H}}_{k,LS}X_{k}\right)}{\partial {\hat{H}}_{k,LS}}=0$$

Thus, the estimated kth subchannel transfer function can be written as follows:

(32) $${\hat{H}}_{k,LS}=X_{k}^{-1}Y_{k}=H_{k}+\frac{n_{k}}{X_{k}}$$

The channel estimation module uses the reference signal R extracted through the cable in the well and the pre-stored reference signal L to estimate the effect of the channel on the signal:

(33) $$\hat{H}=\frac{R}{L}$$

Once an estimate Ĥ of the channel response is obtained, the received signal can be equalized at the receiving end to recover the transmitted signal, letting Y_k_′ be the result after equalization, as shown in the implementation below:

(34) $$\begin{array}{cc} Y_{k}^{\prime } & =\frac{Y_{k}}{\hat{H}}\\ & =\frac{Y_{k}\times (R^{*}\times L)}{||R|{|}^{2}} \end{array}$$

Therefore, the adaptive frequency domain equalizer is able to correct the received signal according to the channel estimation and recover a signal close to the original transmitted signal, and the effect of this correction is mainly reflected in the quality of the constellation diagram.

## 3. Digital Logic Study of Adaptive Downhole High-Speed Communication System

**Figure 6:**
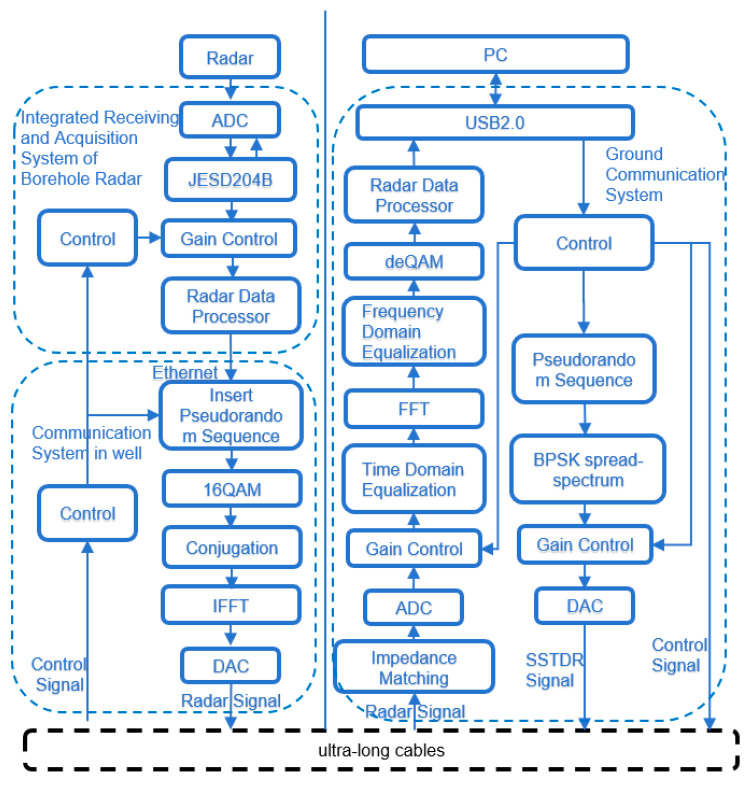
Downhole high-speed communication system.

Digital logic circuits are the basis for the whole system to realize its data interaction, device control, and digital processing, and in this chapter, the design of digital logic circuits in the adaptive downhole high-speed communication system will be discussed in depth. In the beginning, the framework structure of the adaptive downhole high-speed communication system is established by comprehensively analyzing the system requirements in Figure 6. Following this, the chapter explains the digital logic design scheme of each module within the system, covering the implementation details of the adaptive processing technology and its logic control strategy for data exchange with other components within the system.

### 3.1. Digital Logic Overall Programming

#### 3.1.1. Requirements Analysis and Digital Logic Device Selection

This system adopts Xilinx’s Artix-7 series XC7A100T as the device choice of FPGA. Artix-7 series FPGAs are more cost-effective, and the number of its logic resources and pin resources are sufficient to handle the amount of data and the occupied interface part of the communication in the well; its power consumption is also lower. The resources of the XC7A100T are shown in Table 1, and it only needs a reasonable design. The chip can fully meet the requirements of the adaptive downhole high-speed communication system.

**Figure 7:**
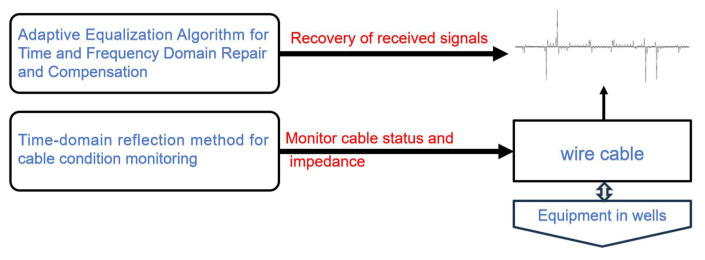
FPGA digital logic architecture design diagram.

Artix-7 chip resources are relatively sufficient to support the adaptive algorithm designed in this system to map digital logic in FPGA. The main goal of the adaptive algorithm module of this system is to accurately receive and repair the high-speed data uploaded from the cable in the well, which guarantees that the equipment in the well has a larger data throughput and more accurate data than before. Among them, the spread spectrum time domain reflection module is used to realize the matching of cable impedance and load impedance, which reduces the loss brought by the cable, and the adaptive equalization algorithm recovers the OFDM data in the well in both time and frequency domains, whose algorithmic function is shown in Figure 7.

#### 3.1.2. System Architecture Design

**Figure 8:**
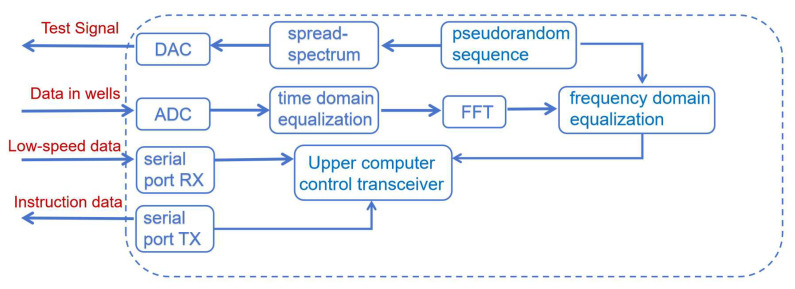
FPGA digital logic architecture overall design diagram.

FPGA is required to take on multiple responsibilities in this system, and the overall design diagram of the structure and interfaces divided according to specific responsibilities is shown in Figure 8:

First of all, it is necessary to map the digital logic of the communication adaptive technology; the algorithm will include the hardware linguistic generation of the equalizer and cable monitor to achieve the recovery of the attenuation signal through the cable channel. Then, it is necessary to communicate with the equipment in the well, with the different communication modes of the equipment in the well using different communication methods, taking into account the high-speed and reliable communication. Also, it is necessary to communicate with the host computer system to select a high degree of adaptability. Finally, to communicate with the host computer system, a highly adaptable plug-and-play communication method is selected to transmit data to the host computer and receive commands from the host computer.

In this system, the digital logic design needs to consider the module reuse and pipeline design and adopt different design schemes for different functional modules. Different from the traditional OFDM, in order to improve the adaptability of the device, this system, the test signal, and the training sequence signal are generated by the pseudo-random sequence generation module; the state of the training sequence signal needs to be taken into account, which should be set to the time domain training signal after OFDM demodulation in the time domain equalization module and to the original unchanged training signal in the frequency domain equalization module. To ensure accuracy, the sequences are pre-stored in the Read Only Memory (ROM) of each module.

#### 3.1.3. Digital Logic Module Functional Planning

**Figure 9:**
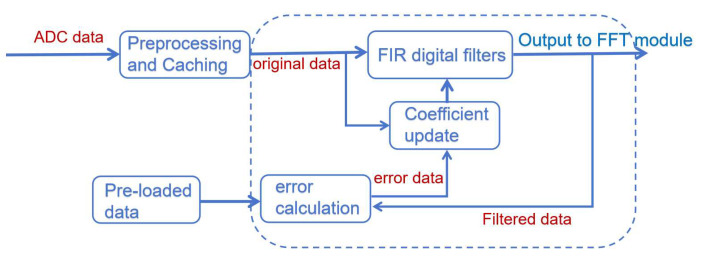
Adaptive time domain equalization module.

**Figure 10:**
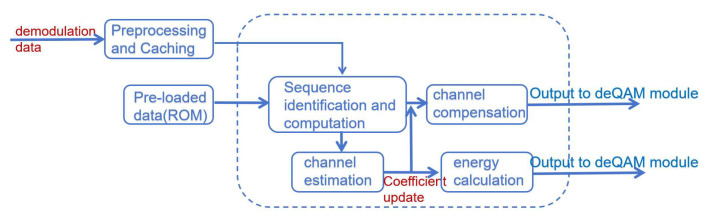
Adaptive frequency domain equalization module.

The digital logic module assumes the core function in this system. This subsection will specify the composition, function, interface, and specific operation scheme of the main digital logic modules:

1. Time domain reflection module: It is mainly composed of the pseudo-random sequence generator and BPSK spreader. The generated data is passed to the DAC control module. This module mainly undertakes the impedance estimation and cable monitoring function of adaptive impedance matching and makes identification of cable status changes.
2. Adaptive time domain equalization module: It is mainly composed of the FIR digital filter, error calculation module, and coefficient update module. It receives data from the ADC back level, while the data generated by the module is passed to the IP core of FFT for OFDM demodulation processing. This module mainly undertakes the function of adaptive time domain equalization to recover the signals from the cable in the time domain. The functional plan of the module is shown in Figure 9.

3. Adaptive Frequency Domain Equalization Module: It is mainly composed of the channel estimation module, energy estimation module, and channel compensation module. It receives data from the FFT IP core, while the data generated by the module is passed to the QAM demodulation module for QAM demodulation processing. This module mainly undertakes the function of adaptive frequency domain equalization and restores the signal from the cable in the frequency domain. The functional plan of this module is shown in Figure 10.

4. Communication interface control module: This includes the AD/DA control module, USB communication module, and serial communication module. These modules are responsible for different functions. Among them, the AD/DA module controls the basic configurations of the ADC and the DAC such as data input and output, working frequency and mode. The AD/DA module controls the basic configuration of the ADC and the DAC such as data input/output, working frequency and mode, and its function is to output and input the test signal of SSTDR and receive the OFDM signal transmitted by the cable in the well; the USB communication module is used to control the working mode of the USB chip and output the high-speed data from the cable in the well to the upper host computer. The serial module is used to parse and encapsulate the UART protocol. It outputs low-speed command data from the host computer to the downhole equipment and receives low-speed data from the rotary encoder of the cable car and passes it to the host computer.

Since the logic design of the communication interface control module needs to be analyzed in conjunction with the specifics of the hardware circuit, this paper will expand the description in Section 4, Hardware Circuit Design.

#### 3.1.4. Test Signal and Training Signal Planning and Design

**Figure 11:**
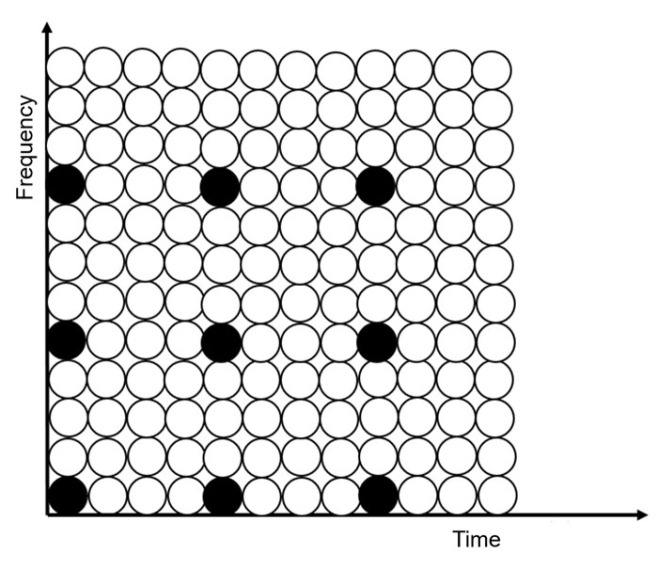
Scattered training sequence distribution map.

OFDM techniques in mobile communications usually use the guide frequency insertion mode, where the training sequence is embedded in the data signal and extracted by the corresponding adaptive recovery module to perform operations to achieve signal recovery [25]. The guided frequency insertion in mobile communication has comb type, block type, and bulk type, and different types have different sensitivities to channel variations and different accuracies. Bulk type training distribution is commonly used in OFDM communication with cables in wells; this type has the highest efficiency in recognizing channel variations in slowly varying channels and is suitable for in-well scenarios in the form shown in Figure 11, where the black color is the training sequence and the white color is the data signal.

**Figure 12:**
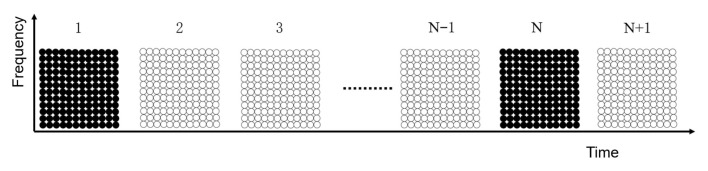
Sequence distribution diagram used in this system.

Distinguished from previous technical solutions, this system innovatively uses a training signal of equal length to the data signal to occupy a complete OFDM signal and receive it at equal intervals. The advantage is that the most complete and accurate channel parameters can be obtained, and the full coverage of subchannel estimation can be realized; the disadvantage is also more obvious, which can only be applied to slowly changing channels. However, the transmit interval can be adjusted according to the actual situation; its sequence distribution is shown in Figure 12, where 1 to N + 1 denotes the first complete OFDM signal to the N + 1th complete OFDM signal, and where the black color is the training signal and the white color is the data signal.

The test signal and the training signal are used as the reference basis for the adaptive technique in this system and are stored in different forms in different modules, in which the test signal generated in the spread spectrum time domain reflection module is generated by the pseudo-random sequence generator and processed as BPSK spread spectrum, and its reference signal is stored in the upper computer, and the inter-correlation operation is placed to be completed by the upper computer, which saves the digital signal processing resources of the FPGA. In adaptive time domain equalization, the training sequence undergoes operations with the same parameters as the IFFT made in the transmitter part and is stored in the ROM inside the module. In adaptive frequency domain equalization, the training sequence is only QAM processed and stored in the ROM inside the module. Among them, the spread spectrum time domain reflection mainly performs the inter-correlation operation on the test signal, while the adaptive time domain equalization and the frequency domain equalization are mainly error calculations, and there are differences in the calculation methods, so the implementation of the algorithms for the different modules needs to be designed according to the actual situation as well.

### 3.2. Spread Spectrum Time Domain Reflection Module Design

#### 3.2.1. Pseudo-Random Sequence Generator Design

The basic principle of the pseudo-random sequence generator lies in the fact that when each clock pulse is obtained, the shift register is shifted to the right by one bit, then the left side of the shift register will be empty by one bit. This generator is called Feedback Shift Register (FSR); if there is an input on the left side, there will be a constant flow of data output from the right output of the shift register. Its structure is presented as some sequence from the shift register which forms the left input according to a certain feedback function.

**Figure 13:**
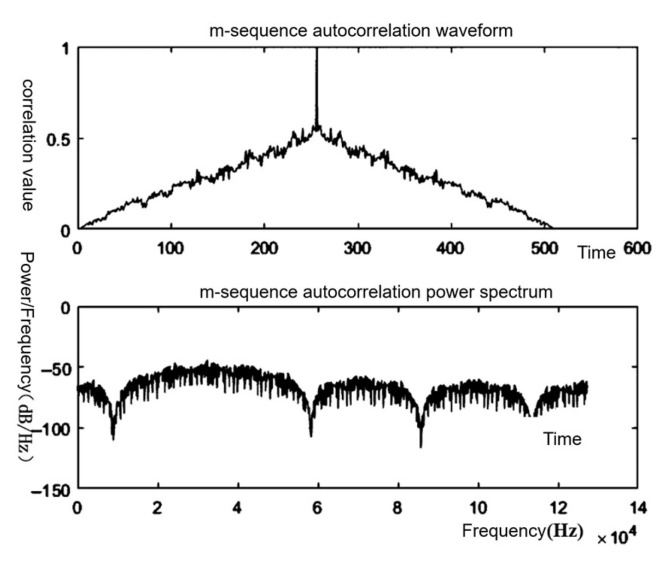
Autocorrelation and power spectrum simulation plots.

In this design, Linear Feedback Shift Register (LFSR) is used as the m-sequence generator, and according to the parameters of the selected m-sequence, the specified number of bits of the register are differentiated and shifted as a whole, thus generating the m-sequence; the simulation effect is shown in Figure 13, which shows that there is a clear sharp peak value.

After determining the parameters, the digital logic circuit of the pseudo-random sequence generator is designed according to the requirements. In this, the generate statement is utilized to generate 64-bit parallel assignment statements to complete the update of each bit of the 64 bits. This design scheme saves larger amount of work.

**Figure 14:**
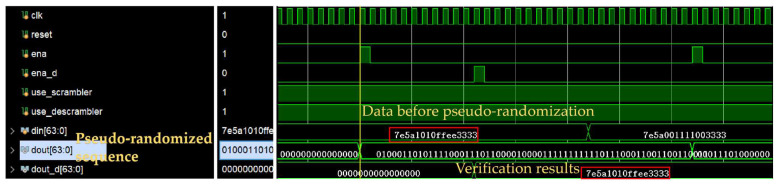
Simulation diagram of pseudo-random sequence generator.

Since the results of the pseudo-random sequence are difficult to confirm the completeness and validity of the sequence by observation, a pseudo-random sequence decoder needs to be designed as a verification tool. By decoding the generated pseudo-random sequence, the final result is obtained as shown in Figure 14. The output result is consistent with the pseudo-randomization before, and it verifies that this is true and valid.

Figure 13 shows the simulation results for the m-sequence: an impulse-like autocorrelation peak at zero lag and an almost flat power spectrum. Figure 14 then verifies the RTL implementation of this sequence. In the testbench, the scrambler uses the same primitive polynomial and seed specified to generate the MLS; the descrambler applies the inverse operation. Therefore, din represents the input test data, dout denotes the converted m-sequence, and dout_d is the test data obtained through inverse processing. It can be observed that dout_d matches din, validating the reliability of the algorithm.

The final result generated has the nature of a random number but is non-random in nature and hence called a pseudo-random sequence. The simulation results also show that the sequence is periodic in nature, and the data is regenerated at the end of the cycle with the previous cycle.

#### 3.2.2. BPSK Spread Spectrum Design

In order to enhance the anti-interference ability of the detection signal, as well as to avoid overlapping with other signals, the pseudo-random sequence is generated and then modulated with BPSK spread spectrum and transmitted into the cable to be tested. After, modulation of the detection signal band is broadened, and at the same time the appropriate frequency band is selected so that it and the cable internal communication signals do not interfere with each other.

**Figure 15:**
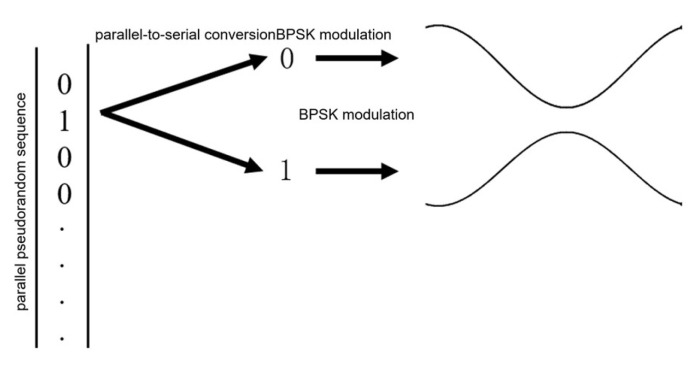
BPSK modulation schematic.

As the pseudo-random sequence used in this system has binary characteristics, the modulation method will be the most appropriate binary digital modulation. From the perspective of the autocorrelation characteristics of the detected signal and the approximate white noise characteristics, our system selects BPSK as the modulation scheme. Its specific implementation is shown in Figure 15.

**Figure 16:**
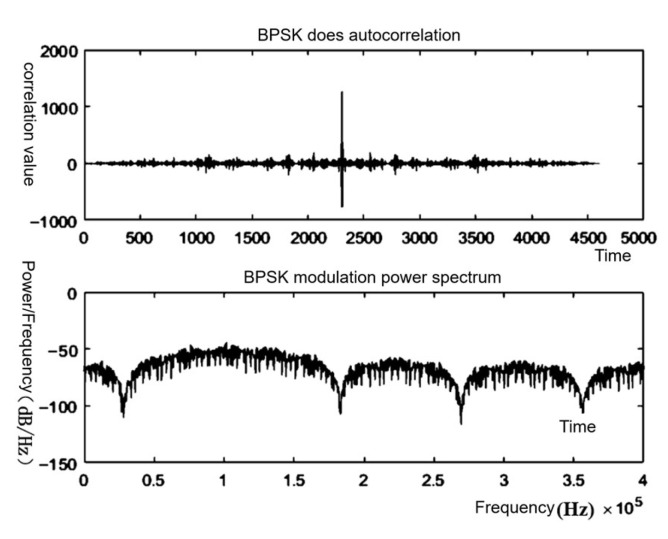
BPSK correlation and power spectrum.

The OFDM modulation and demodulation of this system is chosen to be multielement modulation QAM. The inter-correlation peaks obtained are more pronounced compared to other modulation methods and have better autocorrelation performance and anti-jamming capability. The waveform is shown in Figure 16.

Direct Digital Synthesis (DDS) technique is used to realize BPSK modulation in this design. Its working principle is based on the processing of digital sampling and digital-analog recovery. Xilinx’s FPGA development platforms usually come with DDS IP cores, which are selected from the IP list and called on them. Its detailed parameters are shown in Table 2.

**Figure 17:**
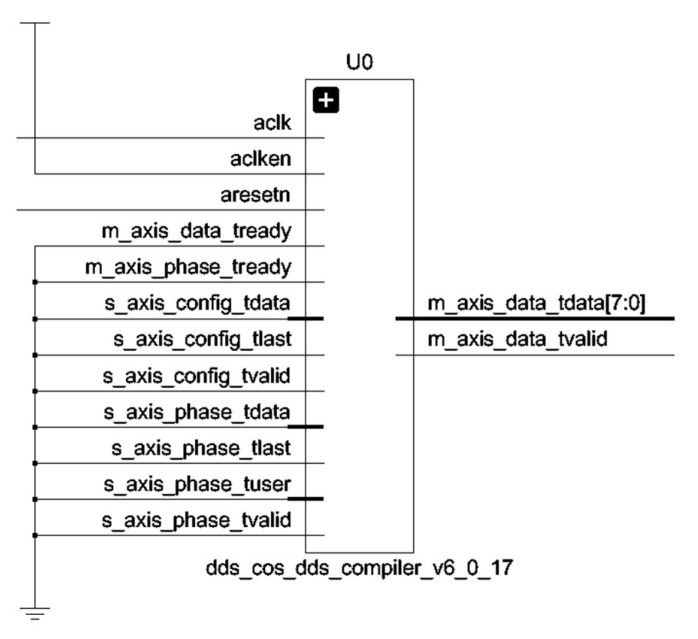
DDS submodule RTL schematic.

In the DDS parameter selection, the architecture selects the mode of the phase generator and trigonometric lookup table. The output frequency is 0.1 MHz, the spurious free dynamic range is 45, the frequency resolution is 0.4, and the output bit width is 8 bits. The control module of DDS is also written, and the generated RTL schematic of the DDS sub-module is shown in Figure 17.

**Figure 18:**
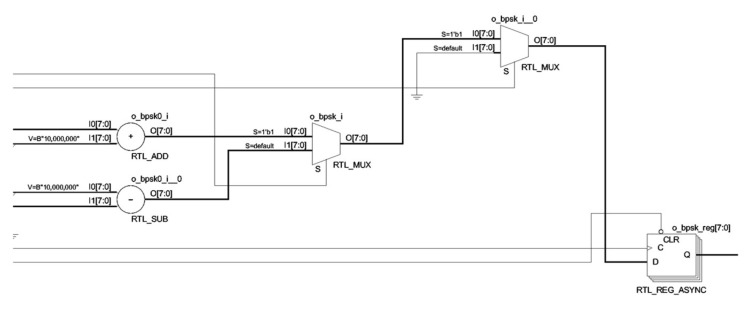
BPSK spread spectrum spreader RTL schematics.

In this system, DDS is used only for BPSK modulation. The control logic of DDS is embedded in the BPSK modulation module for the perspective of readability of digital logic code and resource saving. Its RTL code schematic is shown in Figure 18.

**Figure 19:**
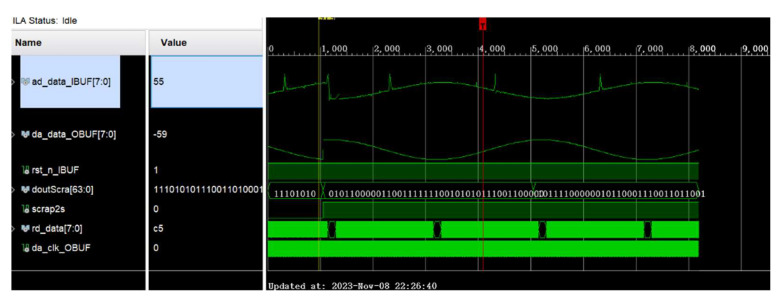
Reflected test signal waveform.

After setting the parameters and the logical control of BPSK, the generated pseudo-random sequence is converted to parallel-serial. The pseudo-random sequence is fed into the spread spectrum module for BPSK modulation in the form of a single bit, which is simultaneously passed through the DAC output and ADC input. An Integrated Logic Analyzer (ILA) is used to capture its real waveform. The modulation effect of the module is shown in Figure 19, where ad_data_IBUF is the captured data.

**Figure 20:**
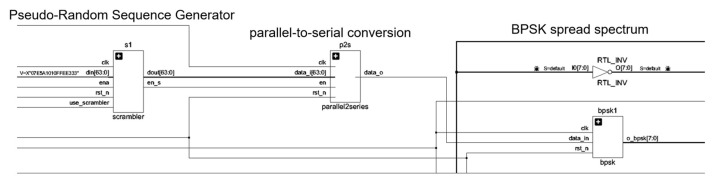
Spread spectrum time domain reflective end RTL schematic.

The DDS module is used as the last link of the spread spectrum time domain reflection method to test the signal generator, and the complete design schematic is shown in Figure 20. The simulation waveform meets the requirements, proving that the digital logic design at the transmitter side achieves the target effect.

### 3.3. Adaptive Time Domain Equalization Design

#### 3.3.1. FIR Filter Design

The communication of this system belongs to the field of low-frequency narrow band wired communication. The FIR filter not only needs to combine with adaptive techniques to equalize the received signals in the time domain, especially to compensate for the attenuated high-frequency part, but it also needs to filter out the high-frequency noise caused by cables and other equipment. After all these considerations, a linear phase FIR filter type IV should be selected.

Considering only the structure of the FIR filter and assuming that its order is 15, the implementation of FIR requires 16 multipliers, which is a large consumption of FPGA resources. However, due to the symmetry of the FIR filter coefficients, it is entirely possible to use a design scheme that multiplies the multipliers. This can greatly save the multiplier resources of FPGA. The filters in this system are designed in parallel. The difficulty lies in the fact that the filter needs to multiply and add 16 sets of time-delayed data in one clock cycle and then output the filtered value.

The parallel design of the FIR filter produces a much smaller delay than the serial design, which is more real-time and meets the basic requirements of high-speed communication. However, the program has higher timing requirements. Considering the stability of data transmission, the design needs to add delay registers at key nodes to meet the timing requirements.

**Figure 21:**
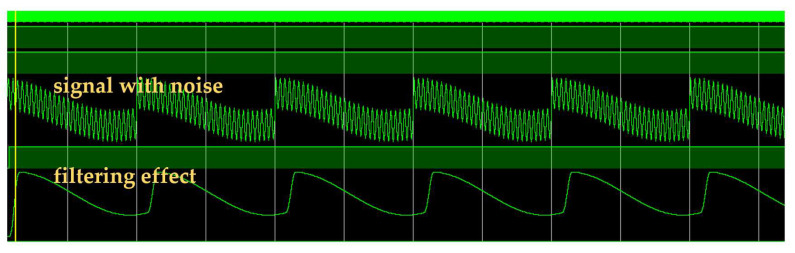
FIR filter simulation diagram.

In order to verify the effectiveness of the designed FIR filter, high-frequency noise with an effective signal frequency of 150 kHz is generated by simulation software. After formal verification, its simulation effect is shown in Figure 21. It can be seen that it has a certain filtering effect.

#### 3.3.2. Design of Error Calculation Module and Coefficient Update Module

The error calculation module needs to recognize the training signals passing through the cable channel and store them in RAM after confirming that they are time domain training signals. The error calculation is performed with the unattenuated time domain training signal stored in ROM.

**Figure 22:**
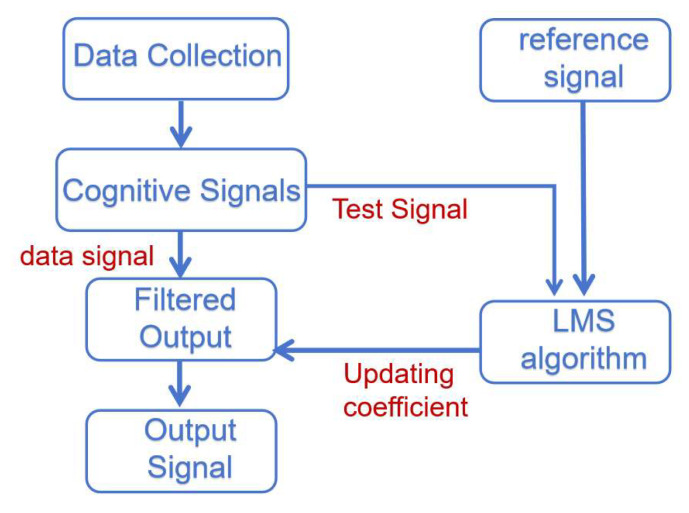
Adaptive time domain equalization schematic.

The computed error input coefficients update module performs LMS operations to obtain new weight coefficients. Then the weight coefficients of the FIR filter are modified. The basic schematic is shown in Figure 22, where the LMS algorithm is specifically implemented in the digital logic language in the following steps: The error difference is multiplied by the received signal and multiplied by the step factor. The resultant output is added to the original weight coefficients to obtain the new weight coefficients. If the input is a data signal, the data signal is filtered and output directly. Where the filter weight coefficients are unchanged from the previous update, other modules are suspended.

**Figure 23:**
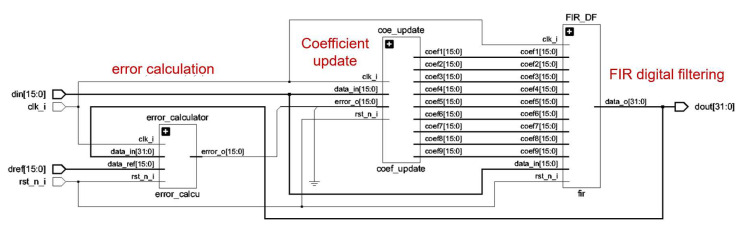
Adaptive time domain equalization RTL schematic.

The overall time domain equalization module consists of three specific sub-modules: the FIR filtering module, the error calculation module, and the coefficient update module. Each submodule is driven by the same clock and reset by the same reset signal. The FIR filter mainly carries out the filtering function, and the error calculation module calculates the error value. The coefficient update module performs the LMS operation. The data inputs of the whole module will go to the coefficient update module and FIR filter module, respectively, and finally output from the FIR filter to the next level after filtering. The schematic diagram of the finalized adaptive time domain equalization digital logic design is shown in Figure 23.

**Figure 24:**
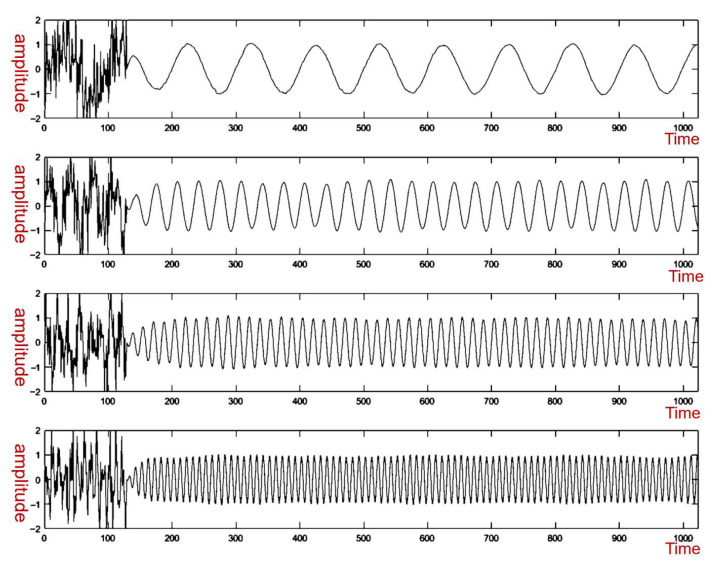
Simulation plots of time domain equalization at different frequencies.

The module is designed and simulated. The simulation software is used to generate the noise triangle signal at different frequencies from 0 to 1 Mhz. The signal enters the module for time domain equalization processing, and the output is then presented by the simulation software. The results are shown in Figure 24, where the picture presents the recovery ability at four different frequencies. It can be seen that the module has a certain recovery ability, but it needs to be gradually corrected to complete the reuse. And before restoration, the waveform changes from small to large.

### 3.4. Adaptive Frequency Domain Equalization Design

**Figure 25:**
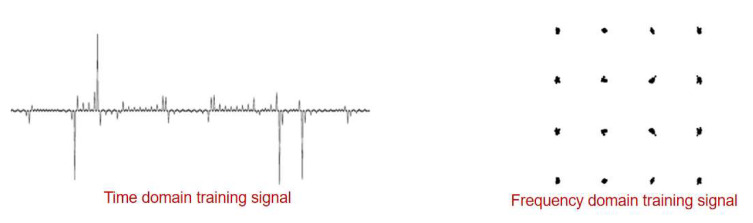
Different forms of training signals.

Frequency domain equalization is usually used in OFDM systems and is performed after the FFT demodulation session. Since the signal has already been through the IFFT at the transmitter side, it needs to be demodulated by FFT transform at the receiver side. Therefore, the training sequence referenced by the frequency domain equalization is the QAM data without IFFT/FFT modulation. Similarly to the adaptive time domain equalization design logic, data is inserted into the data stream by the transmitter to help the receiver in channel estimation. Unlike time domain equalization, the two training sequences have different forms despite the fact that they are essentially the same data. The form is shown in Figure 25.

**Figure 26:**
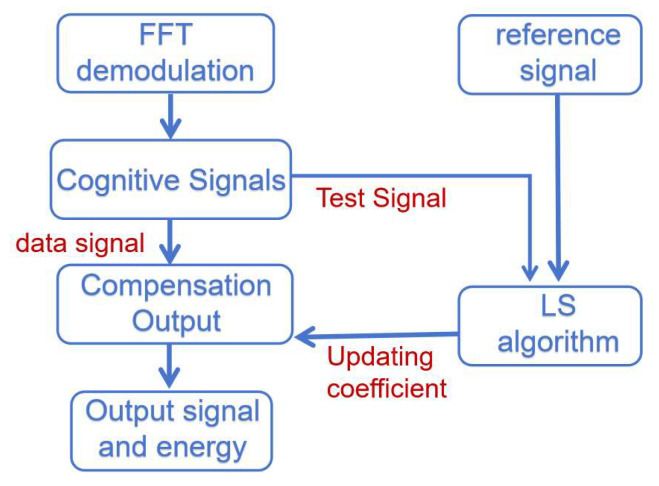
Adaptive frequency domain equalization schematic.

The entire schematic of adaptive frequency domain equalization is shown in Figure 26. It can be seen that its overall thinking is similar to that of adaptive time domain equalization. However, it is worth noting that the module receives the virtual and real signals and operates on the virtual and real signals separately, as well as outputs them.

In the system shown in Figure 26, the LS algorithm plays a central role in the channel estimation module. It provides the subsequent channel compensation module with the data to be compensated. This algorithm directly affects the accuracy of channel compensation and the quality of the final signal. Through this series of processing, the frequency domain equalization can effectively counteract the various channel effects encountered by OFDM signals in the transmission process, thus ensuring the accurate transmission of data. In principle, it is similar to adaptive time domain equalization. If the input is the frequency domain signal of the data, the frequency domain signal of the data is output after compensation. Where the channel compensation coefficients are unchanged from the last update, the channel estimation module is suspended.

**Figure 27:**
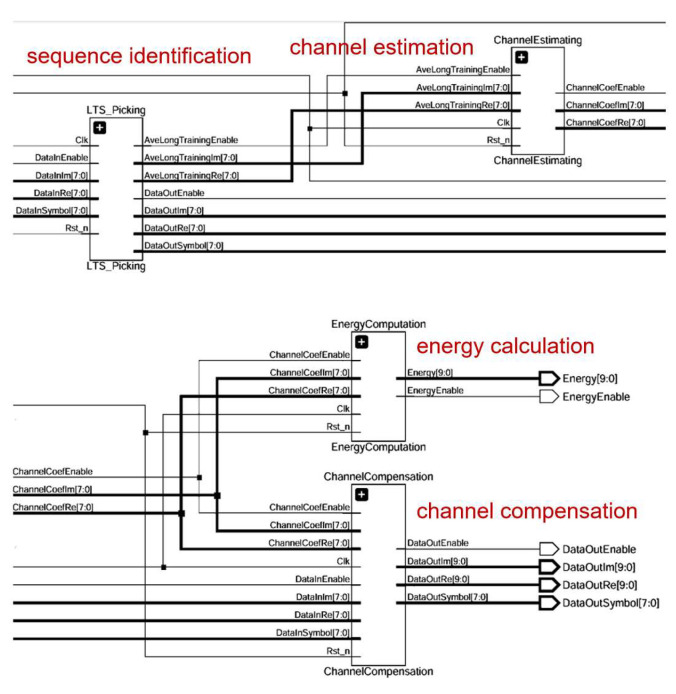
Adaptive frequency domain equalization module RTL schematic.

According to the schematic diagram, the overall module consists of four specific sub-modules: a training sequence identification module, a channel estimation module, an energy calculation module, and a channel compensation module. Each submodule is driven by the same clock and reset by the same reset signal. Among them, the channel compensation module mainly undertakes the function of frequency domain signal recovery. The energy calculation module calculates the signal energy to provide a threshold judgment basis for subsequent QAM demodulation. The channel estimation module performs the LS operation. The RTL design schematic is shown in Figure 27.

The channel estimation module utilizes the extracted training sequences to estimate the effect of the channel on the signal. In this module, the LS algorithm is used to compute an estimate of the channel based on the extracted training sequences and the received signals, implemented by taking the conjugate of the received training signals and multiplying it with the local sequences. With the LS algorithm, the channel response on each subcarrier can be estimated. The energy calculation module obtains the energy distribution of the signal at each frequency point after reception. By analyzing the energy of the received signal on each subcarrier, it is possible to determine which frequencies are more affected by the channel, so that adjustments can be made accordingly during channel compensation.

**Figure 28:**
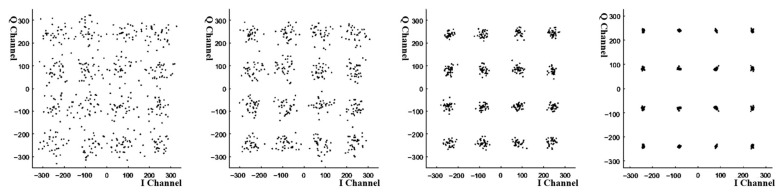
Recovery effect of fading signal at different moments.

With the results of the channel estimation module, the channel compensation module adjusts the amplitude and phase of the received signal to compensate for the effects caused by the channel. A signal as close as possible to the original transmitted signal is recovered. The 16QAM constellation diagram generated by the simulation software is processed with noise addition and high frequency attenuation, and the processed signal enters the module for frequency domain equalization. The equalized data is presented by the simulation software. The specific simulation effect is shown in Figure 28, in which from left to right is the recovery effect of 16QAM constellation diagram at different moments and with the development of time. It can be seen that the frequency domain equalization module has a certain recovery ability.

Unlike the previous time domain equalization, the frequency domain signal generated by the IP core of the FFT contains both real and imaginary paths. The design needs to consider both real and imaginary paths. This is reflected in the energy signal calculation. The squaring operation is performed on the imaginary and real data, respectively, and the LS operation is performed on the imaginary and real signals, respectively, in the channel estimation module.

### 3.5. Spread Spectrum Time Domain Reflectometry Experiment

**Figure 29:**
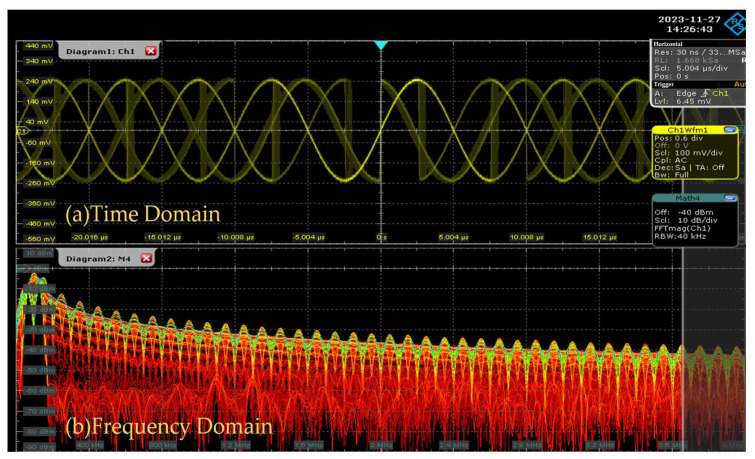
Detected signal waveform and spectrum.

As an adaptive technique that requires transmitting and receiving waveforms, ADC and DAC are essential. SSTDR needs to send a test signal to the cable in the well through DAC and receive the echo through the ADC. To validate the spread spectrum time domain reflection module, the generated pseudo-random sequence is output to an oscilloscope through the DAC. The waveform generated by the spread spectrum time domain reflection method and its spectrogram are shown in Figure 29. It can be seen that the phase of the BPSK waveform generated by the FPGA can correspond to the 1s and 0s of the pseudo-random sequence and that the experimental results are good.

**Figure 30:**
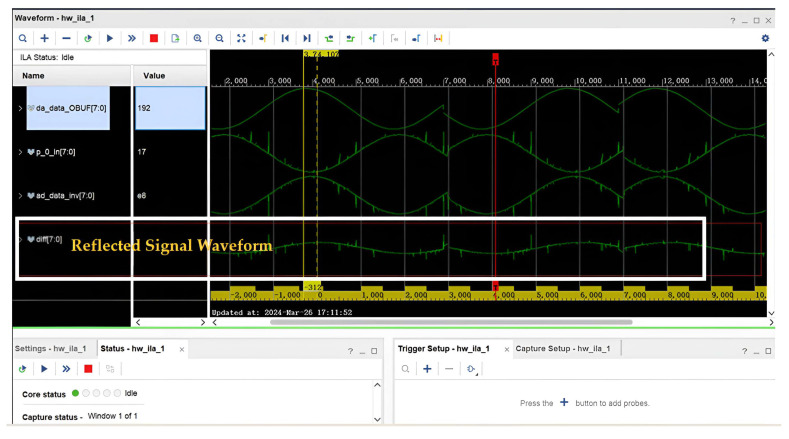
Spread spectrum time domain reflection method reflected signal waveforms.

After validation, the DAC was connected to the ADC with a triple adapter, and a wire was drawn out to connect the start of the cable separately. The reflection waveform generated by the spread spectrum time domain reflection method is shown in Figure 30. It can be seen that the reflected signal waveform is the attenuated waveform of the detection signal emitted by the source. It can also be demonstrated that the SSTDR method, when used in cables, does receive reflected waves and that such reflected waves carry information about the cable channel.

The FPGA resource utilization and power consumption are shown in Table 3. The FPGA meets all performance metrics for resource utilization, power consumption, and real-time performance, with a power consumption of only 1.072 W.

## 4. Verification of Experimental Results

### 4.1. Data Validation

The borehole radar data were compared with Sinopec’s geological drilling logs (GR). Overall, the two datasets agree closely. In particular, the strong reflection attributed to the casing at ~7250 m is consistently observed in both datasets, confirming the system’s detection accuracy in the key stratum. Quantitatively, the GR-curve correlation reaches 0.92, and the depth error for the key bottom marker is 0.013%, demonstrating reliable performance at ~7500 m depth.

**Figure 31:**
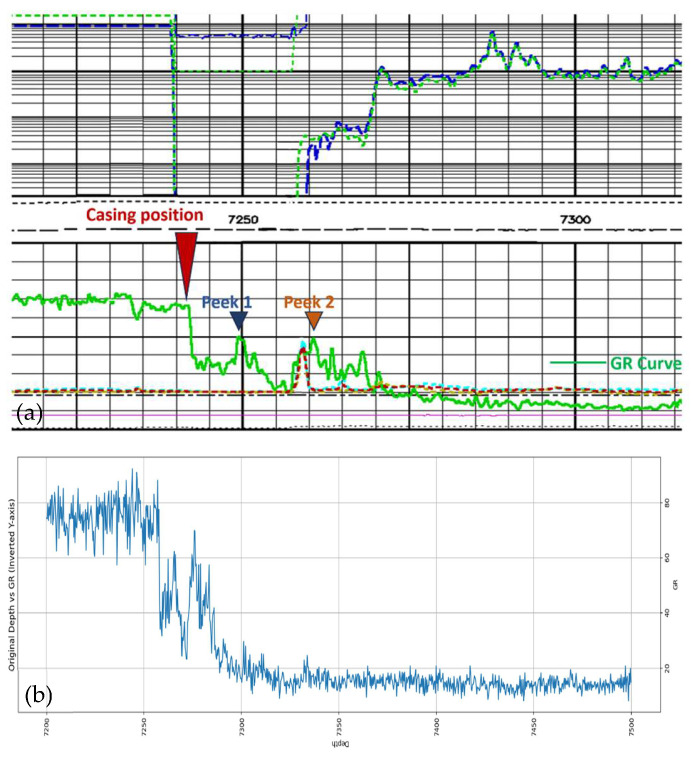

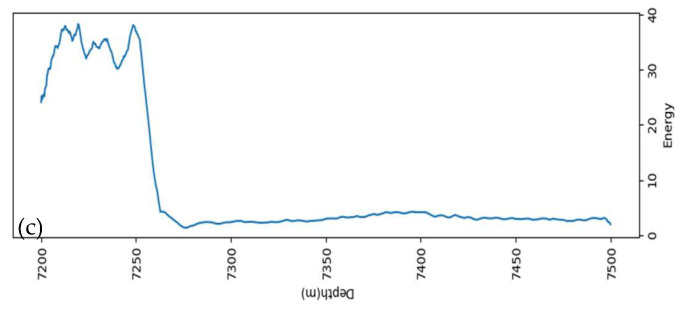
Comparison of third-party data. (**a**) Casing comparison in third-party data, (**b**) measured GR data, (**c**) measured energy data [26].

In Figure 31a, an abrupt change in GR at ~7240 m occurs when the radar exits the casing and enters a sediment mixing zone, producing a sharp GR transition. Corresponding GR discontinuities are also evident at ~7256 m in Figure 31b,c. Because the downhole radar tool length is up to 16 m, a systematic depth offset of ~15 m between the measured radar-referenced depths and third-party log depths is expected. After accounting for this offset, the features align well: in the third-party data (Figure 31a), two small GR peaks appear just beyond the casing (at ~7250 m and ~7261 m), and the measured GR-curve (Figure 31b) shows the corresponding peaks at ~7260 m and ~7283 m. The detailed discrepancies are summarized in Table 4 and are consistent with minor amounts of target material mixed within the sediments, which produce the small GR peaks.

### 4.2. System Performance Evaluation

The upgraded borehole radar system exhibits markedly improved depth reach, interference robustness, and measurement fidelity. Compared with conventional architectures, it resolves fine-scale features of deep geological structures with higher contrast and spatial continuity, thereby enhancing target delineation and improving the efficiency of oil and gas prospecting.

## 5. Conclusions

This work presents, to our knowledge, the first fielded borehole-radar deployment for geological exploration at ~7500 m in the Shunbei area, enabled by a channel-adaptive “communication + acquisition” architecture. The system integrates SSTDR (m-sequence BPSK) for in situ cable/termination monitoring and adaptive matching, time domain LMS equalization for waveform restoration, and frequency domain OFDM equalization based on LS channel estimation, all realized in real time on a cost-effective FPGA. Field results show strong agreement with third-party logs: the GR-curve correlation reaches 0.92, the ~7250 m casing reflector is consistently reproduced, and the key bottom depth error is 0.013%, confirming reliable operation under extreme downhole conditions and improved depth reach, interference robustness, and data fidelity. Although the field test at 7500 m demonstrated a GR-curve correlation of 0.92, this paper lacks discussion on experimental reproducibility, error sources, and performance under varying operating conditions. Looking ahead, we will perform the following: ① Conduct repeat measurements and cross-well comparisons across diverse formations, mud systems, and well depths; ② Integrate multi-sensor logging technologies to broaden application scenarios. Additional wells or numerical simulations are recommended for validation to enhance conclusion generalizability and reliability; ③ Implement multiple repeat runs within the same well to ensure accuracy. We believe these plans will substantially improve the conclusions’ universality and reliability.

## Figures and Tables

**Table 1:** XC7A100T resource table.

IO	Block RAM	DSP Slices	Logic Cells	Slices
284	4860	240	101,440	15,850

**Table 2:** DDS parameter selection.

Configuration Options	Output Frequency (MHz)	Spurious Free Dynamic Range	Frequency Resolution	Output
Phase generator and SIN COS LUT	0.1	45	0.4	[7:0]

**Table 3:** FPGA resource table.

Resource	Temperature	LUT	Total Power	BRAM	IO
**Utilization**	28.4 °C	13203	1.072 W	126	37
**Available**	71.6 °C	101400	/	325	285

**Table 4:** Errors in third-party data comparisons.

Third-Party Data(m)	Measured Data(m)	Length After Radar	Error
7240	7257	7241	0.025
7250	7265	7249	0.05
7261	7285	7259	0.1

## Data Availability

Data are contained within the article.
